# Effectiveness and safety of acupotomy on lumbar spinal stenosis: A pragmatic randomized, controlled, pilot clinical trial: A study protocol

**DOI:** 10.1097/MD.0000000000028175

**Published:** 2021-12-23

**Authors:** Ji Hoon Han, Hyun-Jong Lee, Sang Ha Woo, Yu-kyeong Park, Ga-Young Choi, Eun Sil Heo, Jae Soo Kim, Jung Hee Lee, Chung A. Park, Woo Dong Lee, Chang Sop Yang, Ae-Ran Kim, Chang-Hyun Han

**Affiliations:** a Department of Acupuncture & Moxibustion, College of Korean Medicine, Daegu Haany University, Daegu, Republic of Korea; b Department of Diagnostics, College of Korean Medicine, Daegu Haany University, Daegu, Republic of Korea; c Pre-major of Cosmetics and Pharmaceutics, College of Herbal Bio-Industry, Daegu Haany University, Daegu, Republic of Korea; d KM Science Research Division, Korea Institute of Oriental Medicine, Daejeon, Republic of Korea; e R&D Strategy Division, Korea Institute of Oriental Medicine, Daejeon, Republic of Korea; f Korean Convergence Medicine, University of Science & Technology (UST), Campus of Korea Institute of Oriental Medicine, Daejeon, Republic of Korea.

**Keywords:** acupotomy, lumbar spinal stenosis, pilot, pragmatic, randomized controlled trial

## Abstract

**Introduction:**

Lumbar spinal stenosis (LSS) is a pathological condition that causes a variety of neurological symptoms due to narrowing of the anatomical structures; usually, conservative treatment is recommended, rather than surgical treatment. Acupotomy combines conventional acupuncture with small scalpels; the procedure can be considered minimally invasive, and has recently received considerable attention in clinical practice. Still, there is a lack of data and randomized controlled trials regarding acupotomy related to LSS. Additional studies are necessary, considering the low methodological quality and small size of the study.

**Methods and analysis:**

This is a pragmatic, pilot, randomized controlled trial. The trial comprises 8 weeks of treatment, with 16 visits and a 4-week follow-up period. Forty participants diagnosed with LSS will be randomly assigned to either the experimental or control groups; both groups will receive acupuncture and interferential current therapy twice a week for 8 weeks, while the experimental group will receive an additional acupotomy intervention once a week for 8 weeks. The primary outcome will be assessed using the visual analog scale; the secondary outcome will be measured by self-rated walking distance, Oswestry Disability Index, and short-form McGill Pain Questionnaire. Measurements will be obtained prior to the start of the clinical trial, 4 weeks after the interventional procedure, 8 weeks after the procedure, and 4 weeks after the end of the interventional procedure. Blood tests and adverse reactions will be performed to ensure safety of the treatments.

**Conclusion:**

We expect that this study will provide basic data for future large-scale acupotomy studies regarding LSS.

## 1 Introduction

Lumbar spinal stenosis (LSS) is a pathological state that causes a variety of neurological symptoms. Narrowness of the spinal canal, root canal, or intervertebral foramen—caused by bulging discs, hypertrophy of surrounding bones, and overgrowth of soft tissue that damages the nerve and vascular components—can also cause neurological symptoms.^[1]^ LSS may present as low back pain, hip pain, and lower extremity pain caused by walking, standing, or stretching; other symptoms include discomfort, sensory loss, and weakness of the lower extremities.^[2]^

Exercise, medication, epidural steroid injections, physical therapy, and surgery are well-known treatments for LSS.^[3–6]^ Generally, conservative treatment is recommended, rather than surgical treatment. Although many patients undergo multiple conservative treatments for LSS repeatedly, they often experience the associated symptoms and eventually require surgical treatments such as nerve decompression and spinal fusion. However, patients often experience postsurgical complications and lower satisfaction after these surgical treatments^[7]^; therefore, patients try to find safer and more effective conservative treatments—including acupuncture—for LSS treatment.^[8,9]^ Acupuncture—supported by compelling evidence as an appropriate treatment for chronic pain—is an effective complementary alternative to treatment for spinal stenosis.^[10]^ Consequently, many studies have recently been conducted regarding the various forms of acupuncture, including acupotomy and thread embedding acupuncture, designed to enhance the effectiveness of acupuncture.^[11,12]^

Acupotomy—a type of acupuncture that combines conventional acupuncture with a small scalpel—has recently garnered attention, and can be considered minimally invasive.^[13]^ Acupotomy has received considerable attention in clinical practice; however, there is a lack of data and randomized controlled trials (RCTs) regarding acupotomy related to LSS. Additional studies are necessary considering the low methodological quality and small size of the study.^[14]^ Therefore, we plan to conduct an RCT to evaluate the effectiveness of pain reduction, improvement in quality of life, and safety of acupotomy in patients with LSS.

## 2 Materials and methods

### 2.1 Study design

This is a pragmatic, pilot, RCT, approved by the Daegu Oriental Hospital of Daegu Haany University Clinical Trial Review Committee (DHUMC-D-21001-ANS-01) and registered with the CRIS (KCT0006234). This report complies with the Consolidated Standards of Reporting Trials guidelines. This study protocol complies with the tenets of the Declaration of Helsinki and Korean Good Clinical Practice. The trial comprises 8 weeks of treatment, with 16 visits and a 4-week follow-up period.

### 2.2 Participants

Forty participants diagnosed with LSS will be enrolled in this clinical trial; they will be recruited through advertisements posted in hospitals, the hospitals’ webpages, etc. All participants will be provided with a complete explanation of the trial protocol and will receive a written explanation and informed consent form. Each participant has the right to withdraw the clinical trial; participants will be excluded for violating the protocol, not meeting the inclusion criteria, or withdrawing consent.

### 2.3 Eligibility criteria

The inclusion criteria are: an age of 50 to 80 years; low back pain or lower extremity pain for at least 3 months; LSS diagnosed by magnetic resonance imaging or computed tomography; a visual analog scale (VAS) score between 4 and 7; no problems with language, expression, and concentration; being able to attend follow-up evaluations during the trial; and voluntarily signing an agreement to participate in the study.

Exclusion criteria are: side effects or a history of hypersensitivity to acupuncture treatment; requiring severe surgical treatment for neurological symptoms of cauda equine syndrome, or sensory or motor paralysis; having undergone spinal surgery prior to the clinical trial; having undergone treatment for epidural nerve block within 3 months of the trial; neuromuscular scoliosis or neurodegenerative diseases; results of liver function tests (alanine aminotransferase, aspartate aminotransferase) exceeding 3 times the normal range; abnormal kidney function test results; a neurological or psychologically important medical history, or a related pre-existing condition; pregnant women, lactating women, and women with plans to conceive or who do not agree with appropriate contraception options; taking anticoagulant medication; use of an artificial pacemaker; any participant regarded as inappropriate by the director of the clinical trial.

### 2.4 Randomization and blinding

An investigator with no further involvement in the study generated the allocation sequence of the binary random numbers with a probability of 0.5 for each binary number. The binary random numbers were generated by the “randbetween” function in the Excel program, until the experimental and control groups were equal in size. These successive trials were stochastically independent. The assignment envelope will be sealed and opaque to conceal allocation. During the clinical trials, neither the participants nor clinical practitioners will be blinded; however, outcome assessors will be blinded when they measure outcomes. Participants registered in the experimental group will receive usual care (acupuncture and interferential current therapy [ICT]), and participants assigned to the control group will receive additional acupotomy treatment during usual care.

### 2.5 Intervention

All participants will be randomly assigned to the experimental or control groups, and all treatments will be treated by a Korean medical doctor. Both groups will receive acupuncture and ICT, twice a week for 8 weeks; the experimental group will receive an additional acupotomy intervention once a week for 8 weeks.

#### 2.5.1 Acupotomy

Participants will undergo acupotomy with a 0.50 × 50 mm sterile, disposable, stainless steel acupotomy (DongBang Acupuncture Inc., Gyeonggi-do, Republic of Korea). Acupotomy is performed at 7 pre-defined acupoints (the governor vessel at the corresponding level of stenosis in the imaging diagnosis, bilateral Hyeopcheok acupoints, and upper and lower bilateral Hyeopcheok acupoints) without needle retention time. Additionally, hip tender points around GB30 and tender points of the hamstring muscles can be treated, depending on the participants’ needs. When an invasive intervention is needed, it is inserted slowly while also monitoring whether the participants complain of pain in the neural course.

#### 2.5.2 Acupuncture

Participants will undergo acupuncture with 0.30 × 0.40 mm and 0.35 × 0.60 disposable, sterilized, stainless steel acupuncture needles (DongBang Acupuncture Inc.) at 17 pre-defined acupoints (GV3, and both sides of BL23, BL24, BL25, BL26, GB30, GB31, BL40, and BL60). Additionally, several acupoints (if the radiating area is up to the buttock, GB29; if the radiating area is up to the thigh, BL36 and BL37; if the radiating area is up to the calf, BL55 and BL57) may be further included, considering the participants’ needs. The acupuncture needles will be inserted to a depth of approximately 50 to 60 mm with electrical stimulation, and BL24 and BL26 will be stimulated by electroacupuncture (ES-160; ItoCo. Ltd., Saitama, Japan) at an intensity of 3 Hz. The retention time will be 20 ± 5 minutes.

#### 2.5.3 ICT

ICT (GOODPL Inc., Gangwon-do, Republic of Korea) was performed by attaching 4r parts to the lumbar region of the corresponding level of stenosis in the range of 4000 to 4100 Hz. The intensity (mA) will be set to ensure that the participants do not feel pain, and will be maintained for 15 ± 5 minutes.

### 2.6 Outcome measurement

The primary outcome of this study will be assessed using the VAS.^[15]^ Secondary outcomes will be measured by self-rated walking distance, short-form McGill Pain Questionnaire (SF-MPQ),^[16]^ and Oswestry Disability Index (ODI).^[17]^ Measurements will be obtained prior to the start of the clinical trial, 4 weeks after the interventional procedure, 8 weeks after the interventional procedure, and 4 weeks after the end of the interventional procedure. The treatment schedule and outcome assessments are presented in Table 1.

**Table 1:**
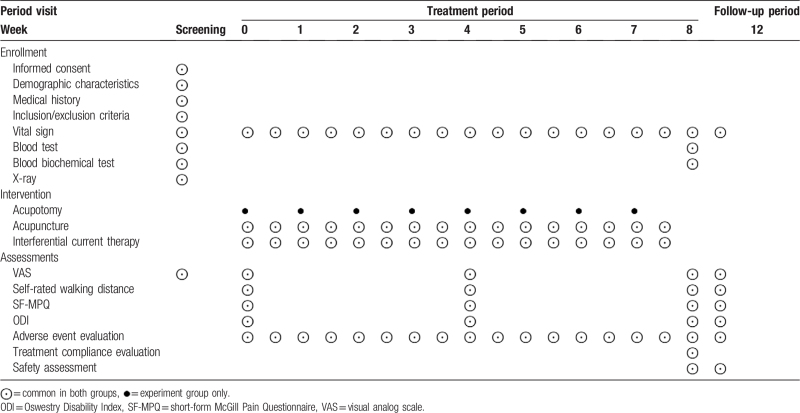
Schedule of the treatment and outcome assessment.

#### 2.6.1 Primary outcome measure

##### 2.6.1.1 VAS

The VAS (from 0–10; 0, no pain; 10, worst pain imaginable) is used to measure subjective pain intensity, which cannot be objectively quantified. The participants indicate the point representing their current pain intensity, and the assessors measure the length from the left side of the horizontal line, to the point indicated by the participants.

#### 2.6.2 Secondary outcome measures

##### 2.6.2.1 Self-rated walking distance

The assessment of self-rated walking distance will be evaluated by the fourth ODI item. In many LSS studies, ODI is measured to investigate the overall function of the patients. The fourth ODI item is also used to quantify individual walking ability, which is known to be the most responsive and valid score of any change in measured walking ability. The fourth ODI item is expressed as a score from 0 to 5; a higher score indicates a lower walking ability.

##### 2.6.2.2 SF-MPQ

SF-MPQ is a short form of the existing MPQ, which is widely used to assess the magnitude of pain experienced. Each item consists of 15 adjectives; these represent the sensory and affective magnitude of pain experienced using a descriptive scale, the Present Pain Index (PPI), and VAS. The 15 descriptive scales include 11 sensory and 4 affective words; the words on the descriptive scale are graded on a 4-point scale (0 = no symptoms, 1 = mild, 2 = moderate, or 3 = severe), while the Present Pain Index scale measures the current pain magnitude on a 6-point scale (0 = no pain, 1 = mild, 2 = discomforting, 3 = depressing, 4 = horrible, or 5 = excruciating).

##### 2.6.2.3 ODI

The ODI is known to be highly valid for evaluating the general living function of patients with a backache, and was found to be highly reproducible in patients with LSS and neurological claudication. The ODI evaluates 10 ordinary items: pain intensity, personal care, lifting, walking, sitting, standing, sleeping, sex life, social life, and traveling; each everyday activity is graded on a 6-point scale. The scores of all the items answered are summed, divided by the number of items answered, and multiplied by 100; a high score means that the level of disability in daily life due to low back pain is high. The verified Korean version of the ODI will be used in this study.

### 2.7 Safety

The safety of this trial will be assessed by the white blood cell count, platelet count, red blood cell count, differential count, hematocrit, hemoglobin level, erythrocyte sedimentation rate, aspartate aminotransferase level, alanine aminotransferase level, blood urea nitrogen level, creatinine level, C-reactive protein, prothrombin time, partial thromboplastin time, serum potassium level, serum sodium level, and serum chloride level. Participants will undergo hematological and blood biochemical tests before, as well as 8 weeks after the procedure, for safety verification.

All adverse events (AEs) and vital signs will be monitored at every visit. In principle, the participants will be asked to immediately report any AEs; if AEs occur during the clinical trial, the association between acupotomy or usual care in clinical trials will be evaluated, and a follow-up investigation conducted.

### 2.8 Statistical analysis

Statistical analyses will be conducted on the basis of the Clinical Trial Statistical Guidelines (KFDA, 2000).

SPSS Win. Ver. 26 (IBM, Armonk, NY) will be used for the statistical analyses.

The statistical package used based on the Clinical Trial Statistical Guidelines (KFDA, 2000) was SPSS Win. Ver. 26 (IBM, Armonk, NY). The last observation carried forward method will be used for missing data due to dropouts. The significance level will be set at 5% for statistical significance. Intention-to-treat analysis will be conducted for all data analyses. The study will confirm the clinical characteristics and comparative equivalence of demographic variables between the experimental and control groups. To assess baseline characteristics of quantitative data between groups, the Student *t* test for the parametric method or Mann-Whitney *U* test for the non-parametric method will be used. The chi-squared (χ^2^) test will be used to compare qualitative data between groups. To compare baseline measurements with those obtained at 4, 8, and 12 weeks regarding VAS, self-rated walking distance, SF-MPQ, and ODI, the paired *t* test (parametric method) or Wilcoxon signed-rank test (non-parametric method) will be used; repeated measures analysis of variance will be conducted to evaluate the differences between groups. If the interaction between the group and time is statistically significant, we will investigate the point where the resulting pattern changes between the 2 groups. The χ^2^-test will be used to compare the incidence of AEs associated with the groups and interventions.

## 3 Discussion

Traditionally, there are several approaches to treating LSS, including both conservative and surgical treatment. It has been reported that the cure rate of surgical and non-surgical treatment cannot be stated conclusively, and that the incidence of side effects increases after a surgery.^[18]^ Therefore, there has recently been a growing interest regarding safer and more effective conservative treatments, such as acupuncture and acupotomy, which have been widely used in clinical practice for the treatment of LSS. Recently, various studies have reported the efficacy and safety of acupuncture and acupotomy in LSS.^[19–21]^

According to the China Association of Acupuncture-Moxibustion's back pain guidelines, acupotomy is recommended for pathological changes in muscles, ligaments, and articular capsules in lumbar soft tissue.^[22]^ Based on a systematic review and meta-analysis evaluating the effectiveness of acupotomy in recently published studies, acupotomy could be clinically beneficial as a minimally invasive treatment for LSS.^[21,23]^

Although the methodological quality of the study is poor and the number of good-quality RCTs few, the superiority of acupotomy has been reported in a meta-analysis.^[21]^ While studies on the efficacy of acupotomy are ongoing, there are limitations to the application of these clinical studies in clinical practice. In real clinical situations, combinations of treatments are utilized more often than single treatments for LSS; pragmatic studies allowing for better decision-making regarding combinations of treatments are therefore drawing attention. Nevertheless, pragmatic acupotomy studies on LSS are rare, and making effective clinical decisions for patients with LSS remains a challenge. Therefore, we designed this pilot, pragmatic study to assess the safety and efficacy of acupotomy guidance in patients with LSS. We expect that the results of this study will provide basic data for conducting future, large-scale acupotomy studies on LSS. Additionally, it is possible to establish a basis for comparing and confirming the effects of adding acupotomy to existing clinical treatments.

## Author contributions

**Conceptualization:** Hyun-Jong Lee, Chang-Hyun Han.

**Data curation:** Ji Hoon Han, Hyun-Jong Lee.

**Formal analysis:** Woo Dong Lee.

**Funding acquisition:** Chang-Hyun Han.

**Investigation:** Ji Hoon Han, Sang Ha Woo, Yu-kyeong Park, Ga-Young Choi, Eun Sil Heo.

**Methodology:** Hyun-Jong Lee, Jae Soo Kim, Jung Hee Lee.

**Project administration:** Ji Hoon Han, Hyun-Jong Lee, Jae Soo Kim.

**Resources:** Chung A Park.

**Supervision:** Hyun-Jong Lee, Jae Soo Kim, Jung Hee Lee, Chang Sop Yang, Ae-Ran Kim, Chang-Hyun Han.

**Validation:** Woo Dong Lee.

**Writing – original draft:** Ji Hoon Han, Hyun-Jong Lee, Jung Hee Lee.

**Writing – review & editing:** Ji Hoon Han, Hyun-Jong Lee, Jung Hee Lee.
